# Does a Graphical Abstract Bring More Visibility to Your Paper?

**DOI:** 10.3390/molecules21091247

**Published:** 2016-09-18

**Authors:** Eva-Maria Pferschy-Wenzig, Ulrich Pferschy, Dongdong Wang, Andrei Mocan, Atanas G. Atanasov

**Affiliations:** 1 Institute of Pharmaceutical Sciences, Department of Pharmacognosy, University of Graz, Universitaetsplatz 4/1, 8010 Graz, Austria; eva-maria.wenzig@uni-graz.at; 2 Department of Statistics and Operations Research, University of Graz, Universitaetsstrasse 15, 8010 Graz, Austria; ulrich.pferschy@uni-graz.at; 3 Department of Pharmacognosy, University of Vienna, 1090 Vienna, Austria; dongdong.wang@univie.ac.at; 4 Department of Pharmaceutical Botany, Iuliu Hațieganu University of Medicine and Pharmacy, 400012 Cluj-Napoca, Romania; mocan.andrei@umfcluj.ro; 5 Institute of Genetics and Animal Breeding of the Polish Academy of Sciences, 05-552 Jastrzebiec, Poland

**Keywords:** graphical abstract, article views, citations, pdf downloads, scientific writing, science communication, research visibility, online attention, social media shares, Altmetric score

## Abstract

A graphical abstract (GA) represents a piece of artwork that is intended to summarize the main findings of an article for readers at a single glance. Many publishers currently encourage authors to supplement their articles with GAs, in the hope that such a convenient visual summary will facilitate readers with a clearer outline of papers that are of interest and will result in improved overall visibility of the respective publication. To test this assumption, we statistically compared publications with or without GA published in *Molecules* between March 2014 and March 2015 with regard to several output parameters reflecting visibility. Contrary to our expectations, manuscripts published without GA performed significantly better in terms of PDF downloads, abstract views, and total citations than manuscripts with GA. To the best of our knowledge, this is the first empirical study on the effectiveness of GA for attracting attention to scientific publications.

## 1. Background 

In the field of publishing, where nowadays the majority of articles are searched for and read online and where scientists increasingly make use of social media platforms to share their findings, many publishers currently encourage authors to prepare a graphical abstract (GA) in addition to the written abstract. The GA is intended to summarize the article´s main findings for readers at a single glance, to attract audience attention, and to make readers pick out one´s article from a plethora of potentially interesting literature. It is also a format that is perfectly suitable for sharing via social media. However, to achieve effectiveness it is optimal to provide a well-designed and highly informative graph. Frequently, authors who do not want to put extra work into a GA simply compile some of the most important figures from their manuscript, a strategy that does not always lead to meaningful GA [1]. It also has to be considered that GA is not equally suitable for all kinds of manuscripts. *Molecules* has been offering the possibility of publishing papers with GA since 2008. 

It should be noted that the effectiveness of GA for attracting attention to publications is frequently claimed by publishers and in Web-blogs. However, a thorough search on Web of Science (Thomson Reuters) did not reveal a single study on this topic. Also, in some of the most relevant journals relating to this issue, such as *Scientometrics*, *Journal of Informetrics* and *Journal of the Association for Information Science and Technology*, we were unable to find any study on the impact of GA on the visibility of publications. This research gap might be due to the difficulty of obtaining relevant data for statistical evaluation.

In order to assess the potential impact of GA, we statistically compared publications with and without GA published in *Molecules* between March 2014 and March 2015, with regards to several output parameters reflecting visibility. The data for statistical evaluation were kindly provided by the *Molecules* editorial office. Statistical evaluation was performed using SPSS, Version 24 (IBM). This seems to be the first study of this kind. Surprisingly, it turned out that the considered sample does not support a positive impact of GA at all. This unexpected finding reflects the need for more extensive studies in the future.

## 2. Data Structure and Descriptive Statistics

In total, 1345 manuscripts were published in the 13 *Molecules* issues released between 1 March 2014 and 31 March 2015. For statistical evaluation, we excluded 19 manuscripts—comments, corrections, concepts, retractions, editorials, and letters—which were manuscript types too rare to be evaluated. Thus, in total, 1326 manuscripts were included in our evaluation, 566 of which were published with GA, and 760 of which were published without GA. About half of the manuscripts were published in special issues (Table 1).

In order to check the data for potential dependencies, two-tailed Chi-square test was performed. The test showed that the distribution of GA is neither independent of the issue type (special or regular issue, *p* = 0.041) nor of the paper type (article or review, *p* = 0.009). More precisely, by applying Fisher’s exact test it can be shown that the ratio of papers with a GA is larger for papers of the “article” type than that for papers of “review” type (*p* = 0.005). Moreover, the ratio of papers with a GA is larger for papers in special issues than for papers in regular issues (*p* = 0.023).

In order to consider potential developments over time, the data were grouped into four quarters (Q1-Q3 covering three consecutive issues each, Q4 covering issues December 2014–March 2015 since issue March 2015 only contained 21 manuscripts; Table 2).

A 2-tailed Chi-square test did not reveal a significant correlation between the time of publication and percentage of GA, indicating that there was no significant increase or decrease in the publication rate of GA within the evaluated period of 13 months.

## 3. Inductive Statistics: Performance Analysis

In order to assess whether the use of GA has an impact on a manuscript´s visibility, we used one-tailed Mann-Whitney-U-Test for comparison of different output parameters (performance indicators) between different groups of manuscripts. The following performance indicators for each manuscript were provided by the *Molecules* editorial office: total number of abstract views, total number of PDF downloads, and total number of citations. Altmetric score—a bibliometric score complementary to traditional citation metrics that is intended to measure the online attention for research output—has been collected manually for each manuscript at https://www.altmetric.com. 

Tested hypotheses, performance characteristics, used manuscript groups and significance levels are summarized in Table 3 (*p*-values > 0.2 are considered not significant).

Interestingly, comparison of manuscripts with or without GA revealed that among all manuscripts, those without GA performed better in terms of PDF downloads, abstract views and total citations (Table 3, Nr 1, 2, 3). This property was highly pronounced in the “journal articles” subgroup (exception: abstract views; Table 3, Nr. 2), and less pronounced in the “reviews” subgroup (Table 3, Nr. 3). This finding is somewhat surprising. One potential explanation might be that GA are more often used by younger scientists who have high affinity to social media. In contrast, more well-established scientists might be less familiar with this rather recent tool, or may be less interested in investing extra work into the preparation of a GA. It is quite likely that articles published by more renowned scientists raise more interest and are more frequently cited, viewed, and downloaded. Another way of explaining this finding could be the direct effect of a well-designed GA which gives the observer a concise idea of the content of the paper and thus makes an explicit abstract view or paper download superfluous. This point of view would suggest that papers with GA might receive fewer but better targeted downloads.

Altmetric scores were not significantly influenced by presence or absence of GA, however, it has to be considered that most of the manuscripts had Altmetric scores of 0 and 1. Therefore, the statistical evaluation of this performance characteristic is of limited value only.

Since we have found that the number of GA differs significantly between regular and special issues, we also evaluated the output parameters for these subgroups. Here, it turned out that among all manuscript types, papers published in special issues had significantly better output characteristics than papers published in regular issues (Table 3, Nr. 5). This effect turned out to be mainly caused by the review articles: review articles were found to be significantly more often included in special issues than in regular issues (see descriptive statistics). In addition, review articles were found to display significantly better performance indicators than regular journal articles (Table 3, Nr. 4). When restricting the evaluated manuscripts to regular articles only, the performance indicators were even slightly better for those manuscripts that were published in regular issues (Table 3, Nr. 6).

Concerning the development of performance indicators over time, unsurprisingly, those manuscripts published earlier had more abstract views, PDF downloads and citations than those published later in the evaluation period (Table 3, Nr.7). Clearly, the given data set covering roughly one year does not permit a more detailed analysis of the effect of GA over time.

## 4. Conclusions and Outlook

To conclude, in our study we could not find an indication that manuscripts with GA are more visible to the scientific community than those without GA. On the contrary, manuscripts without GA published in *Molecules* between March 2014 and March 2015 performed better in terms of PDF downloads, abstract views, and total citations than manuscripts with GA. However, it should be taken into account that our study only covers a limited timeframe and is only restricted to one journal. Larger studies including a larger number of different journals would be necessary to test the universal validity of the pattern that we observed in the analysis presented in this work.

## Figures and Tables

**Table 1:** Structure of the evaluated dataset.

Manuscript Type	Total Number	Published with Graphical Abstract (GA)	Published without GA	Published in Special Issue	Published in Regular Issue
Journal article	1074	478	596	437	637
Communication	37	13	24	14	23
Review article	215	75	140	176	39
Sum	1326	566	760	627	699

**Table 2:** Arrangement of evaluated manuscripts regarding the time of publication.

Time Period	Period	Number of Manuscripts	With GA	Without GA	% with GA
Q1	3/2014–5/2014	259	90	169	34.8
Q2	6/2014–8/2014	350	160	190	45.7
Q3	9/2014–11/2014	366	170	196	46.5
Q4	12/2014–3/2015	351	146	205	41.6
Sum	all	1326	566	760	42.7

**Table 3:** Summary of inductive statistics. * *p* > 0.2 not significant (n.s.).

Hypothesis	Nr.	Restriction to Group	*p*-Values *			
			PDF Downloads	Abstract Views	Total Citations	Altmetric Scores
Without GA > with GA	1	all manuscripts	0.000	0.001	0.012	n.s.
Without GA > with GA	2	journal articles	0.000	0.188	0.066	n.s.
Without GA > with GA	3	review articles	0.196	0.025	n.s.	n.s.
Reviews > articles	4	review+ journal articles	0.000	0.000	0.000	0.000
Special issue > regular issue	5	all manuscripts	0.005	0.000	0.000	0.003
Special issue < regular issue	6	journal articles	0.010	n.s.	0.140	n.s.
Q1 > Q4	7	all manuscripts in Q1, Q4	0.000	0.010	0.003	0.000
