# Erratum: Pferschy-Wenzig, E.-M.; et al. Does a Graphical Abstract Bring More Visibility to Your Paper? *Molecules* 2016, *21*, 1247

**DOI:** 10.3390/molecules21121676

**Published:** 2016-12-06

**Authors:** Eva-Maria Pferschy-Wenzig, Ulrich Pferschy, Dongdong Wang, Andrei Mocan, Atanas G. Atanasov

**Affiliations:** 1 Institute of Pharmaceutical Sciences, Department of Pharmacognosy, University of Graz, Universitaetsplatz 4/1, 8010 Graz, Austria; eva-maria.wenzig@uni-graz.at; 2 Department of Statistics and Operations Research, University of Graz, Universitaetsstrasse 15, 8010 Graz, Austria; ulrich.pferschy@uni-graz.at; 3 Department of Pharmacognosy, University of Vienna, 1090 Vienna, Austria; dongdong.wang@univie.ac.at; 4 Department of Pharmaceutical Botany, Iuliu Hațieganu University of Medicine and Pharmacy, 400012 Cluj-Napoca, Romania; mocan.andrei@umfcluj.ro; 5 Institute of Genetics and Animal Breeding of the Polish Academy of Sciences, 05-552 Jastrzebiec, Poland

The authors wish to make the following change to their paper [1]. The symbol “%” in the last column of Table 2 is incorrect. The numbers are actually ratios so in order to obtain percentages, the numbers should be multiplied by 100.

We apologize for any inconvenience caused to the readers by this mistake. The manuscript will be updated and the original will remain online on the article webpage.
